# Effectiveness of activated carbon masks in preventing anticancer drug inhalation

**DOI:** 10.1186/s40780-016-0062-7

**Published:** 2016-11-03

**Authors:** Junya Sato, Atushi Kogure, Kenzo Kudo

**Affiliations:** 1 Department of pharmacy, Iwate Medical University Hospital, 19-1Uchimaru, Morioka, Iwate 020-8505 Japan; 2 Department of Clinical Pharmaceutics, School of Pharmacy, Iwate Medical University, 2-1-1 Nishitokuta, Yahaba, Iwate 028-3694 Japan

**Keywords:** Activated carbon, Medical mask, Cyclophosphamide, Inhalation, Pleated-type, Stereoscopic-type, Adsorption, Anticancer drug exposure

## Abstract

**Background:**

The exposure of healthcare workers to anticancer drugs such as cyclophosphamide (CPA) is a serious health concern. Anticancer drug pollution may spread outside biological safety cabinets even when a closed system is used. The inhalation of vaporized anticancer drugs is thought to be the primary route of exposure. Therefore, it is important that healthcare workers wear masks to prevent inhalation of anticancer drugs. However, the permeability of medical masks to vaporized anticancer drugs has not been examined. Furthermore, the performance differences between masks including activated carbon with chemical adsorptivity and non-activated carbon masks are uncertain. We investigated activated carbon mask permeability to vaporized CPA, and assessed whether inhibition of vaporized CPA permeability was attributable to the masks’ adsorption abilities.

**Methods:**

A CPA solution (4 mg) was vaporized in a chamber and passed through three types of masks: Pleated-type cotton mask (PCM), pleated-type activated carbon mask (PAM), and stereoscopic-type activated carbon mask (SAM); the flow rate was 1.0 L/min for 1 h. The air was then recovered in 50 % ethanol. CPA quantities in the solution were determined by liquid chromatography time-of-flight mass spectrometry. To determine CPA adsorption by the mask, 5 cm^2^ of each mask was immersed in 10 mL of CPA solution (50–2500 μg/mL) for 1 h. CPA concentrations were measured by high-performance liquid chromatography with ultraviolet detection.

**Results:**

For the control (no mask), 3.735 ± 0.543 μg of CPA was recovered from the aerated solution. Significantly lower quantities were recovered from PCM (0.538 ± 0.098 μg) and PAM (0.236 ± 0.193 μg) (*p* < 0.001 and *p* < 0.001 vs control, respectively). CPA quantities recovered from all of SAM samples were below the quantification limit. When a piece of the SAM was immersed in the CPA solution, a marked decrease to less than 3.1 % of the initial CPA concentration was observed.

**Conclusion:**

The SAM exhibited good adsorption ability, and this characteristic may contribute to avoiding inhalation exposure to vaporized CPA. These results suggest that wearing activated carbon masks may prevent anticancer drug inhalation by healthcare workers.

## Background

Exposure of healthcare workers to anticancer drugs is a serious concern, and may cause health hazards such as carcinogenesis or genotoxicity. These concerns have been supported by several studies, including our report of anticancer drug pollution in a clinical setting, and reports of human exposure to anticancer drugs detected in the urine of healthcare workers [1–4]. Although biological safety cabinets (BSCs) or closed system were used in those cases, environmental pollution was still observed. The main routes of occupational anticancer drug exposure are inhalation, epicutaneous absorption, and ingestion. Inhalation appears to be the main exposure route [5].

Cyclophosphamide (CPA) is the most commonly reported drug as an index of anticancer drug exposure. This is because CPA vaporizes at room temperature (≥23 °C), is carcinogenic and teratogenic, and is one of the most frequently prescribed drugs in cancer chemotherapy [6]. Airborne CPA has been detected in anticancer drug preparation environments [7, 8]. Vaporization can result from CPA droplets scattering during preparation and from spills, disposal of administration equipment, and handling the patient’s excrement. Therefore, use of an N95 mask, or other permeability-resistant mask, is strongly recommended in Japanese guidelines when preparing anticancer drug injections (in particular when a BSC and closed system are not used), cleaning preparation environments or spilled solutions, and crushing or de-capsulating oral anticancer drugs [9]. However, there has been no evaluation of the permeability resistance of medical masks to vaporized anticancer drugs such as CPA. Activated carbon adsorbs various chemicals. We reported that a worksheet containing activated carbon adsorbed anticancer drug droplets scattered during preparation [10]. Therefore, the adsorption ability of the mask containing activated carbon was expected to adequately protect against the vaporized anticancer drug. However, the ability of the activated carbon mask to prevent the permeation of anticancer drugs is not evaluated using similar means to that of a normal mask. This study investigated the permeability of clinical-use masks to vaporized CPA, and determined whether the inhibition of vaporized CPA permeability was attributable to the mask’s adsorptive ability. We also compared three types of masks with varying properties with pleats type or stereoscopic type for a shape, presence of the active carbon.

## Methods

### Materials

Pleated-type cotton masks (PCM) was used Disposable surgical mask (No.433619) manufactured by Hasegawa Menko Co., Ltd. (Nagoya, Japan). Pleated-type activated carbon masks (PAM) was Hopes surgical mask (JM-28C) manufactured by Japan Medical Products Co., Ltd. (Asahikawa, Japan). Stereoscopic-type activated carbon masks (SAM) was used Maskey MD manufactured by Koken Co. Ltd. (Tokyo, Japan). The details of three kinds of masks were indicated in Table 1. CPA was used Endoxan infusion^Ⓡ^ 500 mg manufactured by Shionogi & Co., Ltd. (Osaka, Japan). As for the CPA determination by liquid chromatography time-of-flight mass spectrometry (LC-ITTOF-MS), liquid chromatography mass spectrometry (LC/MS)-grade acetonitrile and distilled water (Wako Pure Chemical Industries, Ltd., Osaka, Japan) were used. As for the CPA determination by high-performance liquid chromatography (HPLC), HPLC-grade acetonitrile and distilled water (Wako Pure Chemical Industries, Ltd., Osaka, Japan) were used. Special grade ethanol (Wako Pure Chemical Industries, Ltd., Osaka, Japan) was used.

**Table 1:** List of experimented mask

Mask type	Pleated type cotton mask (PCM)	Pleated type activated carbon mask (PAM)	Stereoscopic type activated carbon mask (SAM)
Brand name, catalog number	Disposable surgical mask (No.433619)	Hopes surgical mask (JM-28C)	Maskey MD
Photograph			
Material	Polypropylene non-woven paper	Polypropylene and rayon non-woven paper, fiberformed active carbon	Polyester and polyamide filter, fiber-formed active carbon (density of 200 g/m2)
Thickness	0.2 mm	1.0 mm	2.9 mm
Performance test	BFE; 99.0 % PFE; 98.8 %	BFE; 97.4 % PFE; not tested	permeability barrier performance for formaldehyde (0.5 ppm, 40 min) and xylene (20 ppm, 43 min).
Manufacturer	Hasegawa Menko Co., Ltd. Nagoya, Japan.	Japan Medical Products Co., Ltd. Asahikawa, Japan.	Koken Co. Ltd. Tokyo, Japan.

*BFE* Bacterial Filtration Efficiency (%), *PFE* Particle Filtration Efficiency (%)

### Masks

Masks differed with respect to constituent materials (cotton and activated carbon) and mask shape (stereoscopic and pleated). These masks did not conform to the N95 or DS2 criteria (≥95 % prevention performance of 0.1–0.3 μm or 0.06–0.1 μm sodium chloride particle permeability). However, these masks are used frequently in medical practice. Prevention of permeability by the PCM was ≥ 95 %, as evaluated by particle filtration efficiency (PFE, mean particle size 0.1 μm of latex) and bacterial filtration efficiency (BFE, mean particle size 3.0 μm of bacteria), and that of PAM was ≥ 95 %, when evaluated by BFE criteria. The SAM could not be measured by either of these criteria because this mask was designed to prevent the inhalation of organic solvents. However, the material (nonwoven paper) and structure of the SAM were similar to those of the medical N95 mask. The SAM was used to prevent inhalation of formaldehyde in hospital pathology laboratories and in the handling of organic solvents.

### Evaluation of CPA permeability of the mask

**Fig. 1:**
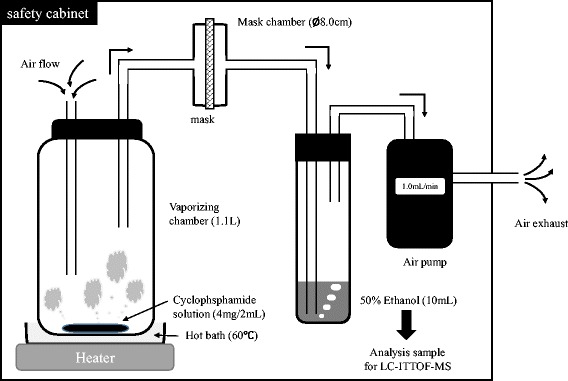
Schematic illustration of experimental equipment for the permeability of the mask for the vaporized CPA. Figure 1 indicated the schematic illustration of experimental equipment for the permeability of the mask for the vaporized CPA. An aqueous solution of CPA (4 mg/2 mL) was vaporized in a chamber (1.1 L) and maintained at 60 °C. The CPA including air passed through three types of masks (100 cm^2^) using an air pump at a flow rate of 1.0 L/min for 1 hour. The air which passed through these masks was recovered in 10 mL of 50 % ethanol solution, and assayed by liquid chromatography time-of-flight mass spectrometry

Figure 1 depicts a schematic illustration of the device used for measuring CPA permeability. The vaporizing method and CPA recovery have been described elsewhere [11, 12]. CPA was used as the anticancer drug and prepared to a solution of 2 mg/mL. The CPA (4 mg/2 mL) was vaporized at 60 °C in a 1.1 L closed chamber. The air containing the vaporized CPA was passed through 100 cm^2^ of each mask using an air pump (MP-Σ3; Shibata Science Co., Ltd., Saitama, Japan). The flow rate was set to 1 L/min, which was the maximal flow of the pump. The aeration and recovery time were set for 1 h, when the quantity of CPA recovered reached a maximum of 4 mg. The mask-passed air was aerated in 10 mL of 50 % ethanol, and the CPA was collected for quantity analysis. Ethanol was used as the recovery medium because CPA readily dissolves in ethanol; however, the ethanol was diluted to 50 % to inhibit vaporization of the ethanol by aeration. The CPA recovery rate under these conditions was around 0.1 %.

### Evaluation of CPA adsorptive ability of the mask

**Fig. 2:**
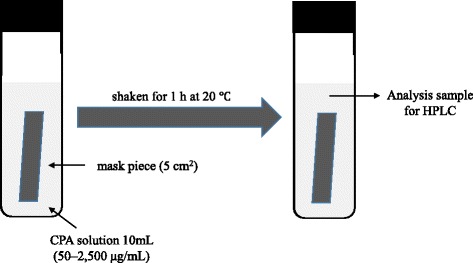
Schematic illustration of CPA adsorptive ability experiment. Figure 2 indicated a schematic illustration of CPA adsorptive ability of the mask. Each mask piece (5 cm^2^) was immersed in various concentrations of CPA solutions (10 mL) and shaken for 1 h at 20 °C. After immersion of one hour, CPA concentration of the solution was measured by HPLC

Figure 2 depicts a schematic illustration of CPA adsorptive ability of the mask. To consider whether the prevention of CPA permeability by each mask was attributable to the adsorptive ability of the mask material, each mask piece (5 cm^2^) was immersed in 10 mL of CPA solution and shaken for 1 h at 20 °C before measuring the CPA concentration. The CPA concentration setting established a concentration gradient (50–2,500 μg/mL) to observe an adsorptive change from low to high concentrations more than we used it for vaporization. After immersion of one hour, CPA concentration of the solution was measured by HPLC.

### CPA measurement

To evaluate CPA permeability for each mask, we measured the quantity of CPA in the recovered solution by LC-ITTOF-MS (Shimazu Co., Kyoto, Japan). For liquid chromatography, 1 μL of the sample was injected into a column (CAPCELL PAK ADME; 5 μm, 2.1 × 150 mm, Shiseido, Tokyo, Japan) which was maintained at 40 °C as a mobile phase at a flow rate of 0.2 mL/min with a 1:1 acetonitrile to water mixture. For mass spectrometry, CPA was ionized by the electrospray ionization (ESI) method, and fragments (m/z = 260.03) were detected in the positive mode. The detection limit was 0.001 μg/mL.

To evaluate the CPA-adsorptive ability of each mask, CPA concentrations in the mask-immersed solutions were determined using HPLC with ultraviolet detection (Hitachi D-2000 Elite system; Hitachi High-Technologies Co., Ltd., Tokyo, Japan) following a previous protocol [13]. Briefly, 20 μL of the sample were injected into a column (LiChrosorb® RP-8; 5 μm, 2.1 × 150 mm, Merck KGaA, Darmstadt, Germany) maintained at 40 °C as a mobile phase at a flow rate of 1.0 mL/min with a 1:3 acetonitrile to water mixture. CPA was measured by ultraviolet absorption at a wavelength of 195 nm. The detection limit was 3 μg/mL.

### Statistical analysis

Measurements were repeated four to five times in each group; numerical values represent the mean ± standard deviation. Differences between the groups were calculated by analysis of variance (ANOVA). If a significant difference was detected, Tukey’s test for multiple comparisons was performed. Hazard ratios lower than 5 % were considered statistically significant. The statistics software used was Excel statistics 2012 (Social Survey Research Information Co., Ltd., Tokyo, Japan).

## Results

### Evaluation of CPA permeability of the mask

**Fig. 3:**
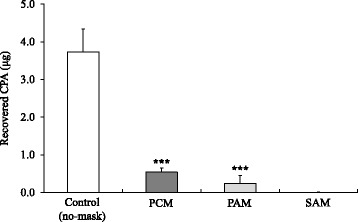
Permeability of the masks for the vaporized CPA (*n* = 5). Fig. 3 indicated the permeability of the masks for the vaporized CPA. The determination in each group was repeated four times. Each bar indicated as mean ± standard □;Control (no mask), ;PCM, ;PAM, ;SAM, respectively. *** indicated a significant difference by Tukey test with less than 0.1 % of hazard ratio as compared with Control. SAM indicated below the limit of quantification

Figure 3 represents the CPA permeability details for each mask. The quantity of CPA recovered in the control group (no mask) was 3.734 ± 0.543 μg. In contrast, use of the mask significantly decreased the quantity of CPA recovered (F_3, 16_ = 143.7, *p* < 0.001 by one-way ANOVA). The CPA quantities recovered from the PCM and PAM were significantly lower (0.538 ± 0.098 μg, *p* < 0.001, and 0.236 ± 0.193 μg, *p* < 0.001, respectively) than those recovered from the control. The quantity of CPA recovered from the PAM was not significantly different from that recovered from the PCM (*p* = 0.483). However, the quantity of CPA recovered from the SAM was below the quantitation limit. Among the five SAM measurements, we failed to detect a peak in two of the measurements, and the other three were below the quantitation limit. Therefore, the quantity of CPA recovered from the SAM was estimated at <0.006 μg.

### Evaluation of CPA adsorptive ability of the mask

**Fig. 4:**
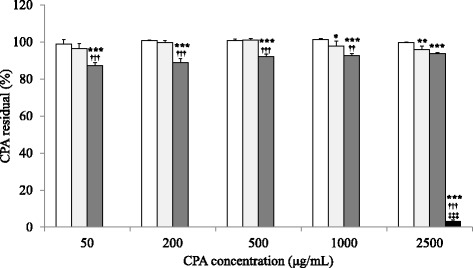
Absorptive ability of the mask immersed in the CPA solution (*n* = 4). Fig. 4 indicated the absorptive ability of the mask immersed in the CPA solution. The determination in each group was repeated five times. Each bar indicated as mean ± standard deviation with □;Control (no mask), ;PCM, ;PAM, ;SAM, respectively. *, **, and *** indicated significant differences by Tukey test with less than 5 %, 1 %, and 0.1 % of hazard ratio as compared with Control, respectively. †† and ††† indicated a significant difference by Tukey test with less than 0.1 % and 1 % of hazard ratio as compared with PCM. ‡‡‡ indicated a significant difference by Tukey test with less than 0.1 % of hazard ratio as compared with PAM. 50–1000 μg/mL in the SAM indicated below the limit of quantification

Figure 4 represents the CPA adsorptive ability of each mask. The results of two-way ANOVA were as follows: CPA concentration, F_4, 60_ = 11.1, *p* < 0.001; Mask, F_3, 60_ = 22,526.3, *p* < 0.001, and CPA concentration × mask interaction, F_12, 60_ = 6.5, *p* < 0.001. CPA concentrations in the PCM-immersed solutions were slightly lower than those of the control. CPA concentrations in the PAM-immersed solutions were significantly lower than those of the control and PCM. However, the decrease was minimal, with 87.2–93.8 % of the initial concentrations remaining. In contrast, CPA concentrations were markedly lower in the SAM-immersed solutions, and lower than the limit of detection at 50–1,000 μg/mL of the initial concentrations. Although CPA was detected at 2500 μg/mL, a significant decrease was found, with only 3.1 % of the initial concentration remaining.

## Discussion

In the previous studies, environmental pollution and human exposure were reported even when a BSCs was used [13]. A pollution survey undertaken in our hospital found environmental pollution of fluorouracil (5-FU) and CPA outside the BSC even though a closed system was used for preparing and handling CPA [1]. For this reason, it was thought that an air barrier might fail upon movement of arms of persons in and out of the cabinet during preparation from Class IIb BSC. It was also thought that anticancer drugs scattered at a preparation would adhere to the infusion surface, and flow out of the BSCs with the infusion bag or bottle [14]. Drugs leaking out of the BSCs would cause human exposure through adhesion to skin or inhalation of the vaporized form. Therefore, wearing a mask is essential.

The particle diameter and physicochemical properties (mist form or molecular form) of the vaporized CPA in this study were unclear. However, a difference in the quantity of recovered CPA was observed when vaporized CPA was filtered through three mask types. The PCM had a ≥ 95 % prevention of permeability, as evaluated by PFE (mean particle size 0.1 μm of latex). The findings implied that the masks trapped most of the CPA, including particles with a ≥ 0.1 μm diameter. The quantity of CPA that permeated the mask decreased by 85.6 % compared with the amount detected under conditions without the mask, even with the PCM. Therefore, wearing the mask might be effective, to some extent, in reducing the inhalation of the anticancer drug droplets. However, some of the CPA filtered through the mask and was recovered in this study. The diameter of the vaporized CPA, in either molecular or particle form, may have been smaller than the captured range of the mask. This suggests that inhalation of vaporized CPA cannot be prevented completely with a normal surgical mask. A similar phenomenon was reported in a study evaluating the reactive oxygen species (ROS) permeability of various masks. Removal efficiencies of particle-form ROS were 83.5–94.1 %, but those of gaseous ROS were only 1.3–21.1 % [15]. The quantity of CPA recovered from the SAM implied very low permeability to CPA (no peak detection in two of five samples, and below the quantitation limit in the other three). This good performance of the SAM may have resulted from trapping of gaseous CPA, as well as particle-form CPA, by the activated carbon. To assess this possibility, we immersed mask pieces in aqueous CPA solutions and examined the change in CPA concentration. The masks containing activated carbon (PAM and SAM) decreased CPA concentrations in the solution to a greater extent than PCM, which did not contain activated carbon. These differences appear to be attributable to the activated carbon. In particular, SAM showed stronger CPA adsorption than PAM. This difference is thought to contribute to the anti-permeability property against vaporized CPA. The density of activated carbon in SAM was high (200 g/m^2^). Although the density of activated carbon in PAM was not specified by the manufacturer, the thickness of the activated carbon was smaller macroscopically than that of the carbon in SAM. Anticancer drugs have been detected in masks used by healthcare workers engaged in preparing the drugs [3, 4]. Therefore, the drug adsorptive ability of the activated carbon mask would seem to be a beneficial factor in preventing anticancer drug exposure in healthcare workers.

This study had some limitations. The use of a mask with N95 standard or superior performance is recommended in Japanese guidelines [9]. However, the three mask types used in this study were not examined with regard to the N95 criteria. Generally, the filtration efficiency of pleats-type surgical masks, for particles with >0.1 μm diameter, is approximately 20–30 % lower than that of the N95 mask [16]. Furthermore, a leak may result from a gap between the face and the mask, depending on mask design and appropriate use by the healthcare worker, subsequently affecting filtration efficiency. If the mask does not fit well, its filtration efficiency is decreased as air bypasses the filter. Rengasamy et al. reported that leaks were greater in pleats-type masks than in stereoscopic-type masks [17]. However, in this study condition, the mask which cut to 100 cm^2^ was pinched with two funnels, and was ventilated as a flat shape. Therefore, it was thought that the difference of the mask shape was not reflected in this results. The effectiveness of preventing anticancer drug inhalation for the clinical application might be lower than this study results. In addition, our study has limitations concerning experimental conditions. The prevention of drug permeability by the mask might be due to the low recovery rate of 50 % ethanol. Although a considerable amount of CPA may penetrate the mask, the ability of the mask to prevent drug permeation might have been overestimated as by low CPA recovery rate. It is uncertain whether the medical practice environment in which anticancer drugs are prepared and administered was correctly replicated in this study. The respiration rate of a human at rest is 8–11 L/min (https://unit.aist.go.jp/riss/crm/exposurefactors/documents/factor/body/breathing_rate.pdf. go.jp /riss/crm/exposurefactors/); the air flow in our study was carried out at one-tenth of that rate. When the airflow is faster, the adsorption of gaseous CPA might decrease because the drug’s contact time with activated carbon is shorter. Moreover, permeability was only evaluated for 1 h. This may be considered short compared to mask exchange cycles in clinical settings. With prolonged use, CPA may filter through the mask. We selected CPA because it is easy to volatilize and is a strong anticancer drug. The ability of anticancer drugs such as ifosfamide and CPA to vaporize and pollute clinical settings has been known [11, 18]. In addition, 5-FU and methotrexate have been detected in hospital air samples [18]. Future studies should investigate permeability of the mask for drugs other than CPA as well.

The CPA occupational exposure limit has been estimated at 0.1 μg/m^3^/day [19]. A healthcare worker is estimated to inhale approximately 3840–5280 L of air over the course of 8 h. Though the bioavailability of inhaled CPA is unknown, the CPA concentrations permitted in healthcare workers with normal body surface (1.73 m^2^ are estimated at 0.03–0.05 ng/L. CPA pollution in studies of hospital air samples were reported as <0.04–10.1 ng/L [7] and 0.24 ng/L [8]. Inhalation without a mask in a clinical polluted environment might to exceed daily CPA exposure limits. SAM decreased the CPA permeation quantity by >99.8 % compared with that under conditions without the mask. When SAM is used in a medical environment similar to what may occur during inhalational CPA exposure, the inhaled CPA may be expected to less than the daily exposure tolerance limit. Therefore, the use of activated carbon masks may be a better choice than the use of other types of masks.

This study was the first to report the examination of the permeability of medical masks to aerosolized anticancer drugs. Furthermore, the performance differences between masks containing activated carbon and normal masks were examined. The mask containing a particular kind of activated carbon (SAM) showed good prevention of permeability. The ability of the activated carbon to adsorb the anticancer drug was suggested as an underlying mechanism of its effectiveness. In future studies, we expect the development of the N95 criteria for medical masks containing activated carbon. It may be necessary to evaluate performance of activated carbon mask with N95 criteria by monitoring urinary levels of anticancer drugs or their metabolites in healthcare workers.

## Conclusion

The use of activated carbon masks to healthcare workers might prevent anticancer drug inhalation.

## Abbreviations

BFE: Bacterial filtration efficiency
CPA: Cyclophosphamide
PAM: Pleated-type activated carbon mask
PCM: Pleated-type cotton mask
PFE: Particle filtration efficiency
ROS: Reactive oxygen species
SAM: Stereoscopic-type activated carbon mask
